# A High Percentage of Beef Bull Pictures in Semen Catalogues Have Feet and Lower Legs that Are Not Visible

**DOI:** 10.3390/ani5030370

**Published:** 2015-07-14

**Authors:** Marcy K. Franks, Temple Grandin

**Affiliations:** Department of Animal Science, Colorado State University, 350 W. Pitkin St., Fort Collins, CO 80523, USA; E-Mail: mkfranks606@gmail.com

**Keywords:** bulls, cattle, hoof, leg conformation, post-legged, semen catalogue

## Abstract

**Simple Summary:**

When cattle breeders purchase semen from a website, the only way they can visually appraise a bull’s conformation is by looking at his photograph. Correct foot and leg structure is important to help reduce lameness. Only 19.4% of the bull pictures on four major websites had fully visible feet and lower legs. A possible explanation for this may be deliberate covering of feet and legs with photo editing software to cover up conformation defects. Visibility of feet and lower legs would help semen buyers avoid bulls with obvious feet or leg problems.

**Abstract:**

A total of 1379 beef bull pictures were surveyed to determine visibility of feet and legs from four American semen company websites. Five different breeds were represented: Angus, Red Angus, Hereford (polled and horned), Simmental, and Charolais. In addition to visibility, data on other variables were collected to establish frequencies and correlations. These included breed, color, material that obscured visibility, such as grass, picture taken at livestock show or outside, semen company, photographer, video, and age of bull. A foot and leg visibility score was given to each bull picture. Only 19.4% of the pictures had fully visible feet and legs. Both the hooves and dewclaws were hidden on 32.5% of the pictures. Correlation between bull’s birthdate and the first four visibility scores was statistically significant (*P* < 0.0001). As age increased the feet and legs were more likely to be visible in the bull’s picture. This may possibly be due to greater availability of both photo editing software and digital photography. One positive finding was that 6% of the bulls had a video of the bull walking which completely showed his feet and legs.

## 1. Introduction

Anecdotal reports from producers indicate that lameness may be increasing in beef cattle. Lameness is a major wellbeing issue because it is a painful condition [1,2,3,4]. It also inhibits a bull from performing essential functions, such as foraging and inseminating cows. Foot and leg conformation problems are related to the occurrence of lameness [2,5,6,7]. Some of these problems include legs that are too straight (post-legged) or hocks that are excessively angled (sickle-hocked). An increase in structural foot and leg conformation problems may be one reason for an increase in lameness. Poor foot and leg conformation and clinical lameness are related to decreased foot angle [1,7,8] and sickled-hocked cattle [7,9] was associated with increased clinical lameness. Claw shape measurements indicated that there is a high association with the occurrence of claw disorders and lameness [10,11,12,13]. In 2015, the second author observed groups of purebred Black Angus finished cattle from a feedlot. These animals had been bred for high marbling and carcass traits and many of them were lame with foot and leg conformation issues. This is why it is important for cattle breeders to select breeding bulls with correct foot and leg conformation to lower the risk of lameness and ensure the welfare of their cattle. To assist in selection for sound feet and legs, ranchers need to be able to see the feet and legs of the animals they select for breeding. This is especially important when selecting breeding bulls from semen catalogues, where visual appraisal of the bull is dependent on the picture supplied by the bull breeder. The objective of this study was to survey beef bull pictures from major semen company websites to determine the visibility of feet and legs. In an increasingly globalized market, it is important for semen buyers to get accurate information. A secondary objective of this study was to detect if there are any other variables that are related to hoof and leg visibility.

## 2. Experimental Section

### 2.1. Sample

In this study, no animals were used and data was collected from websites that were publically available. In 2014, a total of 1379 bull photographs from four major American semen company websites were evaluated for this study. All the bulls from the breeds of Black Angus, Red Angus, Hereford (polled and horned), Simmental, and Charolais were evaluated. The four semen company websites had overlap where the same bull was featured on more than one website. A list of each bull’s name was kept to ensure that the same bull was not scored twice. For data analysis all bulls were identified with a number to keep the bull’s identity confidential. Bulls listed on a website without photos were excluded from this study. 

### 2.2. Foot and Leg Visibility Scoring Method

Visibility of feet and legs were scored on a scale from one to five. Description of each visibility score is listed in Table 1. 

**Table 1:** Foot and leg visibility score of beef breeding bull photographs on semen company websites.

Score	Description
Score 1 Score 2 Score 3 Score 4 Score 5	Legs, hooves, and dewclaws fully visible All four hooves obscured All four hooves and dewclaws obscured All four lower legs (up to the brisket), dewclaws, and hooves obscured Either front or rear feet or legs obscured

### 2.3. Data Collected on Each Bull

Each bull’s breed was included into the dataset. The bull’s coat color was divided into five different color patterns: solid black, black and white, solid red, red and tan, or solid tan. On images that had a visibility score of two or more, the substrate that caused the obscurity was classified as grass, mud, straw/bedding, or snow. Whether the bull was photographed at a livestock show or outside on a ranch was also noted. Occurrence of videos that included the bulls walking was recorded. The pictures that included a photographer’s name were also entered into the dataset. The birthdate published with the bull’s picture was used to give an age of the bull, as of 1 October 2014. Bull’s birthdate was included as a continuous variable. Bulls with no birthdate (*n* = 91) were excluded from this analysis. 

### 2.4. Statistical Analysis

Summary statistics of visibility scores were calculated by SAS [14]. The function PROC FREQ of SAS considered each class variable’s relationship with visibility using a chi-square test. The seven class variables were material obscuring the feet or legs, breed of cattle, coat color, location of picture, semen company website, bull video, and photographer. Spearman’s test was used to look at the continuous variable birthdate for correlation to visibility using PROC CORR of SAS. Visibility score five was dropped from this analysis in order for visibility to be analyzed as an ordered variable of increasing foot and leg cover, since a visibility score of five does not conform to a continuum of ordered scores. Statistical significance was stated as *P* < 0.05.

## 3. Results and Discussion

### 3.1. Foot and Leg Visibility Score

**Figure 1:**
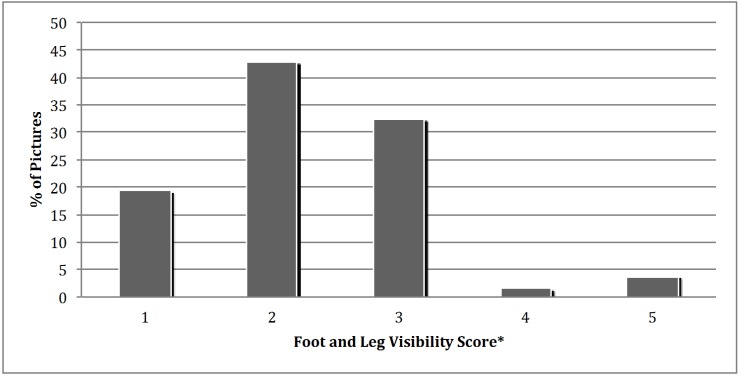
Percentage of beef bull pictures (*n* = 1379) with different visibility scores of feet and legs (*: Visibility Scoring System: 1 = hooves fully visible, 2 = hooves obscured, 3 = hooves and dewclaws obscured, 4 = all four legs covered to brisket, and 5 = either front or back hooves obscured).

The percentages of bulls with different foot and leg visibility scores are shown in Figure 1. Only 19.4% of the bull pictures had full visibility of feet and legs (visibility score one). Therefore, when beef breeders go online to buy semen, in approximately 80% of the pictures, the costumer is not able to see the entire bull. It is important to see the leg down to the hoof, because leg conformation traits are heritable [15]. Not all foot problems can be seen in a picture, but some can be detected without examining the bottom of the foot. For example, corkscrew claw is known to be a heritable condition [16] and can be detected in a picture, especially if the animal is photographed at a front angle. 

All except for two bulls (99.9%) listed on the websites included one side view photograph. This side view photograph is the only way to visually appraise the bull unless a video is included. Locomotion scores evaluating lameness show a high association with foot angle, feet, legs, and rear leg set [6,17,18]. Full visibility of feet and legs would enable a semen buyer to see some of these problems. Including images of bulls photographed at different angles would also help detect additional foot and leg conformation issues. The additional views that should be taken along with the traditional side view pose should be a rear view and front view. These additional photos may show conformation problems that would be harder to detect in a side view, such as corkscrew claw and hooves turning outward. 

One positive finding is that 6% of the bulls had a video of the animal walking. This video is a good way to help the buyer see the bull’s conformation. In addition a video can be reviewed numerous times to ensure nothing is overlooked. 

### 3.2. Data Collected on Each Bull

**Figure 2:**
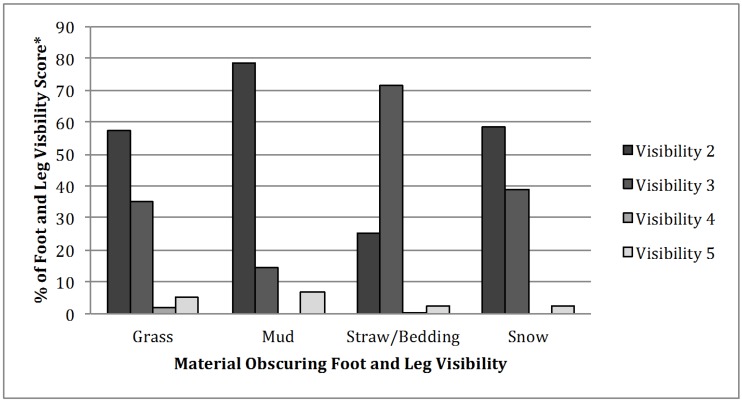
Frequency of material obscuring foot and leg visibility with visibility score on beef breeding bull websites (*: Visibility Scoring System: 2 = hooves obscured, 3 = hooves and dewclaws obscured, 4 = all four legs covered to brisket, and 5 = either front or back hooves obscured).

The frequency of each variable’s relationship to visibility is shown in Table 2. The variable of photographer was dropped from analysis due to missing 769 missing data points. When analyzing the substances that covered up the feet and legs, the visibility score 1 was dropped, because the whole bull was visible. Removing the visibility score one from the frequency test still left a large sample of pictures for materials obscuring visibility and the chi-square test showed it to be significant (*P* < 0.0001) (Table 2). The majority of feet and legs were hidden by grass (80.9%), which is understandable as majority of pictures were taken outside on a ranch (95.5%). A small percentage of the pictures featured bulls standing in mud (1.3%) and nearly 80% of those pictures scored a 2 in visibility (Figure 2). Snow and mud had no scores of visibility four (Figure 2). Over 70% of pictures with obscured hooves and dewclaws were covered by straw/bedding resulting in a visibility score of three (Figure 2). The straw or bedding was stacked high and covered the dewclaws. 

**Table 2:** Chi-square test of frequency of foot and leg visibility to material obscuring foot and leg visibility, breed of cattle, coat color, location of picture, semen company website, and video of bull walking.

Variable	No. of Pictures	*P*-Value
Material obscuring foot and leg visibility Breed of cattle Coat Color Location of picture Semen company website Video of bull walking	1112 1379 1379 1379 1379 1379	<0.0001 0.4484 0.2519 <0.0001 0.1010 0.8129

**Figure 3:**
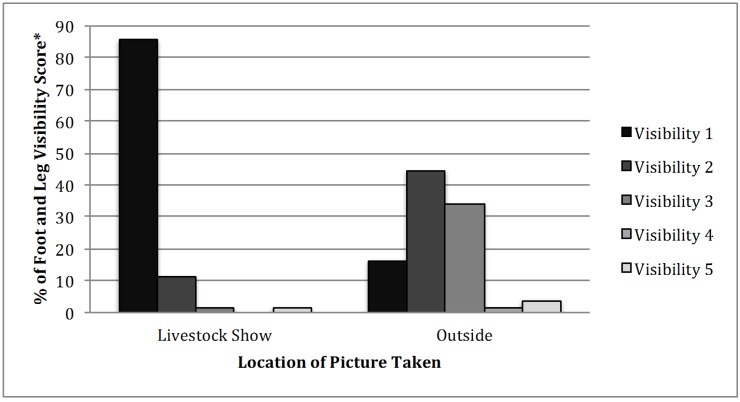
Frequency of location of breeding bull picture taken with visibility score (*: Visibility Scoring System: 1 = hooves fully visible, 2 = hooves obscured, 3 = hooves and dewclaws obscured, 4 = all four legs covered to brisket, and 5 = either front or back hooves obscured).

Also statistically significant was the location of the photograph taken (*P* < 0.0001) (Table 2). Even though only 4.5% (*n* = 62) of the pictures were taken at a livestock show, 85.5% of these images had fully visible legs, dewclaws, and hooves (Figure 3). Pictures taken at shows are taken in front of a backdrop with the name of the show and the floor or ground is usually lightly covered with wood shavings.

Breed, coat color, semen company website, and whether the bull was video taped had no significant relationship with the degree of foot and leg visibility (Table 2). Another variable that was considered for the survey was presence of photo editing. It was not numerically determined which bull pictures had been altered with photo editing software, because accurate detection of photo editing is usually difficult to do. Many images had suspicious signs of being digitally manipulated to obscure the visibility of the feet and legs. One example of noticeable photo editing was tall grass covered the feet and legs and the grass behind the bull was very short. If photo-editing software was used to purposefully hide the bull’s feet and legs, it might have been used to hide conformation problems. 

**Figure 4:**
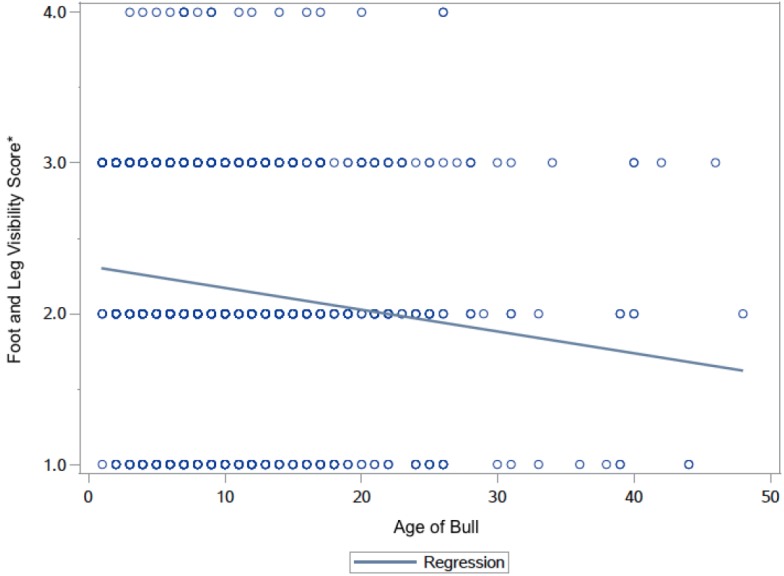
Correlation of age of bull (*n* = 1288) in years and foot and leg visibility score 1–4. Circles with darker outlines indicate a higher count of bull pictures per data point (*: Visibility Scoring System: 1 = hooves fully visible, 2 = hooves obscured, 3= hooves and dewclaws obscured, and 4 = all four legs covered to brisket).

The bull’s ages ranged from one-year-old to 48-years-old. Though many bulls were deceased, age was recorded to provide an approximate time frame for when the picture would have been taken. The correlation between age and foot and leg visibility was highly significant (*r* = –0.169, *P* < 0.0001). The older bulls, which are highly likely to have older pictures, had a higher percentage of animals with visible feet and legs (Figure 4). Some older bull pictures were taken before photo-editing software was readily available. The popular picture-editing program Adobe Photoshop was released in 1990 [19], but many professional photographers were still using film through the mid-2000s. The conversion to digital photography during the last fifteen years makes it much easier to use photo-editing software. Image editing would be an easy way to conceal a bull’s poor foot and leg conformation, instead of posing a bull in tall grass or straw. The easy availability of both digital photography and photo editing software may possibly explain the increase in the percentage of bull pictures with hidden hooves or legs. Another possible explanation might be that present day bulls have a higher rate of structural problems than bulls in the past. This might motivate breeders to cover bull’s feet and legs. This paper warns both bull semen buyers and people interested in animal welfare that photographs of animals may not accurately represent them. Today, easy-to-use photo editing software makes it easy to alter pictures. Both authors have looked at many recent cattle magazines. There are some advertisements for breeding bulls that have more obvious signs of photo manipulation than the semen company websites we surveyed for this paper. 

## 4. Conclusions 

The results of the study show that photographs of beef bulls on major semen company websites have a high percentage with obscured hooves, dewclaws, or legs. Anecdotal reports from producers indicate that beef cattle may be starting to get some of the same foot and leg conformation issues as dairy cattle. It is essential that bull pictures clearly show the animal’s hooves and legs. Increasing the frequency of videos and supplying multiple images of one animal at different angles would be another way to improve the transparency of online evaluation of bulls. Future research should be done with another survey quantifying visibility of feet and legs to determine if a higher percentage of bull pictures will have fully visible hooves and legs. 
